# Do psychiatric disorders affect patient reported outcomes and clinical outcomes post total hip and knee arthroplasty?

**DOI:** 10.1177/20503121211012254

**Published:** 2021-04-29

**Authors:** Sahil Kooner, Jeremy Kubik, Saboura Mahdavi, Sophie (Ghashang) Piroozfar, Hoa Khong, Kanwal Mohan, Eldridge Batuyong, Rajrishi Sharma

**Affiliations:** 1 Department of Surgery, University of Calgary, Calgary, AB, Canada; 2 Alberta Hip and Knee Clinic, Calgary, AB, Canada; 3 Alberta Bone and Joint Health Institute, Calgary, AB, Canada; 4 Department of Psychiatry, University of Calgary, Calgary, AB, Canada; 5 McCaig Institute for Bone and Joint Health, Alberta Hip and Knee Clinic, Calgary, AB, Canada

**Keywords:** Psychiatric, depression, total, arthroplasty, hip, knee

## Abstract

**Introduction:**

The purpose of this study is to evaluate the role of major psychiatric illness on patient outcomes after total joint arthroplasty.

**Methods:**

Patients with a diagnosis of a major psychiatric disorder undergoing total joint arthroplasty were retrospectively matched one-to-one with a cohort without such a diagnosis. Major psychiatric disorder in the registry was identified by diagnosis of anxiety, mood, or a psychotic disorder. Primary outcome of interest included perioperative Western Ontario and McMaster Universities Osteoarthritis Index (WOMAC). Secondary outcomes included EuroQol-5D, adverse events, length of stay, 30-day readmission, and discharge destination.

**Results:**

Total number of patients were 1828. The total hip arthroplasty (37.80 ± 17.91, p = 0.023) and the total knee arthroplasty psychiatric group (43.38 ± 18.41, p = 0.050) had significantly lower pre-operative WOMAC scores. At 3 months, the total hip arthroplasty (76.74 ± 16.94, p = 0.036) and total knee arthroplasty psychiatric group (71.09 ± 18.64, p < 0.01) again had significantly lower 3-month post-operative WOMAC score compared to the control groups. However, outcomes at 1 year were difficult to interpret, as patients with major psychiatric conditions had an extremely high loss to follow-up. Compared to the control groups, the total hip arthroplasty and total knee arthroplasty psychiatric group had an increased length of stay by 1.43 days (p < 0.01) and 0.77 days, respectively (p = 0.05). Similarly, the psychiatric groups were discharged directly home less often (total hip arthroplasty 86.9%, p = 0.024 and total knee arthroplasty 87.6%, p = 0.022) than the control groups.

**Conclusion:**

Patients with the diagnosis of a major psychiatric illness have an increased length of stay and are more likely to require a rehabilitation facility, compared to the control groups. Arguably, of utmost importance, there is a very high rate of loss to follow-up within the psychiatric groups. As such, we recommend these patients should be treated for their diagnosis prior to total joint arthroplasty. Furthermore, importance of clinical follow-up should be emphasized carefully.

## Introduction

Total joint arthroplasty (TJA) procedures are commonly used to treat patients with end-stage arthritis. From 1999 to 2008, the rate of total knee arthroplasty (TKA) in the United States nearly doubled.^1^ By 2030, it is expected that demand for TKA will have increased by as much as 673%, with a similar trend expected for total hip arthroplasty (THA).^2^ Although TJA is a clinically proven and cost-effective procedure, there remains a significant proportion of patients, especially among those who have undergone TKA, that remain unsatisfied with their post-operative outcomes.^3,4^ In fact, patient-reported outcomes have remained relatively stagnant despite advances in implant technology and surgical technique.^2,5^ Pre-operative functional status, patient demographics, medical comorbidities, perioperative adverse events, and variations in surgical techniques do not entirely account for patient dissatisfaction.^6,7^

We are now beginning to understand that outcomes post-TJA are determined by a number of patient-specific factors that may not be directly related to the surgery itself, including pre-operative expectations and psychological health.^8^ Recent evidence suggests that pre-operative psychological comorbidities may negatively impact patient-reported outcomes. Furthermore, unrecognized or undertreated psychiatric conditions may be more common than previously thought, and can also result in poor outcomes after TJA.^9–11^ The prevalence of depression in the TJA patient population is approximately 25%, whereas the prevalence of overall psychiatric illness in orthopaedic inpatients may range from 19% to 86%.^12–14^ Patients with end-stage arthritis may be at higher risk of developing mental illness compared to the normal population as they often experience chronic pain, tend be of older age, and have numerous medical comorbidities.^15^

Given the relatively high prevalence of psychiatric illness and related risk factors in the TJA population, there has been increased interest on understanding how these two conditions affect each other. Therefore, the purpose of this study is to evaluate the role of major psychiatric illness on outcomes after TJA using the data available in the Alberta Bone and Joint Health Institute (ABJHI) registry. We hypothesize that patients with major psychiatric illnesses will report inferior outcomes after TJA compared to those without any psychiatric illness.

## Methods

After institutional ethics approval was granted, we retrospectively collected data from the ABJHI database to identify patients who had undergone primary THA or TKA with a minimum 1 year follow-up between 2013 and 2018. Exclusion criteria included unicompartmental knee arthroplasties, hip resurfacings, simultaneous TJAs, revision surgeries, TJA for infection, and TJA for periprosthetic fracture.

To calculate the sample size, we looked at detecting the mean difference of over five points on the primary outcome (WOMAC). In order to reach α = 0.05 and power of 0.8, a minimum of 143 patients per group (286 in total for TKA and 286 in total for THA) was required. To increase the power of the study and strengthen our study results, we expanded the sample size to 828 THA patients and 1000 TKA patients.

From this cohort we identified our psychiatric group, which included patients that also had a formal diagnosis of a major psychiatric disorder (50% TKA and 50% THA). Major psychiatric disorder was identified in the ABJHI database by determining if the patient had been formally diagnosed with any of the following conditions: bipolar disorder, anxiety disorders, major personality disorder, chronic mental health diagnoses, psychosis, and moderate depressive episodes. The diagnosis of psychiatric illness was captured in the medical history of patient using Clinical Risk Group data (CRG) from 3 years prior to their surgery admission date. These patients were matched one-to-one with patients without such diagnosis. Patients were matched by surgery type, year of surgery, age, gender, and medical comorbidities.

Due to the limitations of our database, the most reliable method of capturing and including comorbidities in our analysis was as an aggregate category versus individual diagnoses. An independent and blinded assessor completed matching for both groups.

The primary outcome of interest included patient-specific, self-reported outcomes, which was measured using validated scoring questionnaire of Western Ontario and McMaster Universities Osteoarthritis Index (WOMAC). Patient outcome was defined pre-operatively and post-operatively at 3 months and 1 year using the Western Ontario and McMaster Universities Osteoarthritis Index (WOMAC) and EuroQol-5D (EQ-5D) scores, and total change in score was measured by finding the difference between the pre-operative score and 12-month post-operative score.

The WOMAC score is a 24-question validated, joint-specific tool that evaluates three dimensions, including pain (5 questions), stiffness (2 questions) and physical function (17 questions).^16^ The ABJHI database reports WOMAC as a range from 0 to 100 (transformed WOMAC score; WOMAC All transformed score = (96-raw score) × 100/96, –Range of WOMAC All transformed score is from 0 (the worst) to 100 (the best), –Range of WOMAC All raw score is from 0 (the best) to 96 (the worst)) with zero indicating total disability and 100 indicating no disability. Therefore, the lower WOMAC score in our results represents the higher disability.

Similarly, the EQ-5D is a valid and standardized instrument for quantifying overall health status.^17^ ABJHI uses a five-level EQ-5D (EQ-5D-5L) version which consists of the EQ-5D descriptive system and the EQ visual analogue scale (EQ VAS). The descriptive system defines health status in five dimensions: mobility, self-care, usual activities, pain/discomfort and anxiety/depression. Each dimension has five categories: no problems, slight problems, moderate problems, severe problems and extreme problems.^18^ The EQ VAS records the patient’s self-rated health on a vertical visual analogue scale, where the endpoints are labelled ‘The best health you can imagine’ and ‘The worst health you can imagine’. The VAS can be used as a quantitative measure of health outcome that reflects the patient’s own judgement.^18^ The ABJHI has copyright permission and authorization to use both WOMAC and EQ-5D. ABJHI is also registered with *EuroQol* group.

Secondary outcomes included early medical and mechanical adverse events, infections, length of stay, 30-day readmission rates and final discharge destination. All patient information was anonymous and de-identified.

### Statistical analysis

Descriptive statistical analysis was completed by comparing control and psychiatric groups for both THA and TKA cohorts. Mean and standard deviation for continuous variables, and the frequency and proportion for categorical variables, were calculated to characterize and compare both groups. Statistical comparison of patient characteristics was performed using two-tailed student t-test and chi-square to detect statistically significant differences at 95% confidence level to ensure both groups were comparable at baseline. Univariate and mixed-effects linear regression were used to examine changes in the EQ-D5 and WOMAC scores. Length of stay (LOS) was calculated as the cumulative number of days spent in hospital between both groups. Cumulative LOS was log transformed and multivariate linear regression analysis was performed comparing cumulative log-transformed LOS for both groups. Risk adjustment factors in the regression model included BMI, sex, and number of pre-surgery risk comorbidities. Statistical analysis was completed with significance defined as p < 0.05. All statistical analyses were performed using the programme STATA version 13 (STATA, College Station, Tex) software.

## Results

In total, 1828 patients were included in our analysis. In THA subset, we included 414 patients in each group (n = 828). These groups otherwise had similar baseline demographics (Table 1). In the TKA subset, we included 500 patients per group (n = 1000). These groups also had similar baseline demographics (Table 2).

**Table 1.** THA group baseline characteristics.

Group	Psychiatric diagnosis	No diagnosis	p value
N	414	414	
Age (average ± SD)	67.3 ± 11.7	67.5 ± 11.3	0.78
Gender (% female)	62.8	62.8	0.78
Comorbidities			0.99
0	55	55	
1	93	93	
2	78	78	
3	63	63	
4	61	61	
5	29	29	
6	22	22	
7	8	8	
8	4	4	
9	1	1	

**Table 2.** TKA group baseline characteristics.

Group	Psychiatric diagnosis	No diagnosis	p value
**N**	500	500	
Age (average ± SD)	66.7 ± 9.7	66.7 ± 9.6	0.99
Gender (% female)	62.8%	62.8%	0.99
Comorbidities			0.99
0	49	49	
1	59	59	
2	78	78	
3	112	112	
4	79	79	
5	56	56	
6	44	44	
7	14	14	
8	9	9	

Regarding the follow-up rate, both THA and TKA cohorts saw minimal loss to follow-up at the 3-month period, but significant loss to follow-up at the 1-year period. For the THA WOMAC cohort, only one pair was lost to follow-up at 3 months, while 378 pairs were lost to follow-up at 12 months, indicating a 91% loss to follow-up rate (LFR). The THA EQ5D cohort demonstrated a similar trend with 32% LFR (58 out of 182 pairs) at 3 months and 87% LFR (159 out of 182 pairs) at 1 year. The TKA WOMAC cohort demonstrated one percent LFR (4 out of 500 pairs) at 3 months and 93% LFR (464 out of 500 pairs) at 1 year. The TKA EQ5D cohort demonstrated 1% LFR (2 out of 195 pairs) at 3 months and 90% LFR (174 out of 195 pairs) at 1 year.

With regard to patient-specific, self-reported outcomes (WOMAC and EQ-5D), both the THA (37.80 ± 17.91 vs 40.74 ± 19.3, p = 0.023; Table 3) and TKA psychiatric groups (43.38 ± 18.41 vs 45.45 ± 20.07, p = 0.050; Table 4) had significantly lower pre-operative WOMAC scores compared to their respective control groups. At 3 months, this trend continued, as both the THA (76.74 ± 16.94 vs 79.16 ± 16.19, p = 0.036) and TKA psychiatric group (71.09 ± 18.64 vs 75.92 ± 16.22, p = 0.00) again had significantly lower 3-month post-operative WOMAC scores. This indicated worse outcomes compared to their respective control groups. The difference between pre-operative and 3-month post-operative WOMAC scores were significantly worse in the psychiatric TKA cohort (27.6 vs 31.6; p = 0.038), although this trend was not true for the THA cohort (p = 0.888; Table 5).

**Table 3.** THA outcomes.

Group	Psychiatric diagnosis	No diagnosis	p value
WOMAC (mean ± SD)			
Pre-operative	N = 414 37.80 ± 17.91	N = 414 40.74 ± 19.32	**0.023**
3 months post-operative	N = 413 76.74 ± 16.94	N = 413 79.16 ± 16.19	**0.036**
1 year post-operative	N = 36 80.16 ± 19.96	N = 36 79.90 ± 19.35	0.950
EQ5D (mean ± SD)			
Pre-operative	N = 182 0.36 ± 0.26	N = 182 0.45 ± 0.22	**0.011**
3 months post-operative	N = 124 0.76 ± 0.17	N = 124 0.81 ± 0.15	**0.002**
1 year post-operative	N = 23 0.78 ± 0.19	N = 23 0.85 ± 0.13	0.170
Adverse event (%)			
Mechanical (%)	4 (1.0%)	9 (2.2%)	0.263
Medical (%)	2 (0.5%)	8 (1.9%)	0.107
Transfusion (%)	60 (14.5%)	51 (12.3%)	0.360
30-day re-admission (%)	16 (3.9%)	19 (4.6%)	0.600
Infections (%)	0 (0%)	0 (0%)	0.999
Discharge home (%)	359 (86.9%)	379 (91%)	**0.024**
Length of stay (mean, days)	6.41 ± 8.37	4.98 ± 4.49	**0.003**

Significance is defined as *p* < 0.05. *p*-value less than 0.05 shows the statistical significance and they have been bolded.

**Table 4.** TKA outcomes.

Group	Psychiatric diagnosis	No diagnosis	p value
*WOMAC (mean ± SD)*			
Pre-operative	N = 500 43.38 ± 18.42	N = 500 45.45 ± 20.07	**0.052**
3 months post-operative	N = 496 71.09 ± 18.64	N = 496 75.92 ± 16.22	**<** **0.001**
1 year post-operative	N = 36 69.17 ± 26.37	N = 36 75.93 ± 20.82	0.230
*EQ5D (mean ± SD)*			
Pre-operative	N = 195 0.47 ± 0.27	N = 195 0.49 ± 0.44	0.488
3 months post-operative	N = 193 0.73 ± 0.19	N = 193 0.78 ± 0.0192	0.170
1 year post-operative	N = 21 0.70 ± 0.23	N = 21 0.79 ± 0.25	0.220
*Adverse event (%)*			
Mechanical	1 (0.2%)	1 (0.2%)	0.999
Medical	13 (2.6%)	23 (4.6%)	0.127
Transfusion (%)	56 (11.2%)	39 (7.8%)	0.057
30-day re-Admission (%)	34 (6.8%)	29 (7.8%)	0.520
Infections (%)	4 (0.8%)	2 (0.4%)	0.340
Discharge home (%)	438 (87.6%)	460 (92.0%)	**0.022**
Length of stay (mean, days)	6.00 ± 7.00	5.23 ± 6.18	**0.050**

Significance is defined as *p* < 0.05. *p*-value less than 0.05 shows the statistical significance and they have been bolded.

**Table 5.** The difference between pre-operative and 3-month WOMAC score.

*THA*			
	Psych	Non-psych	p value
Difference between 3-month and pre-op WOMAC score (Median)	40.70	40.15	0.888
*TKA*			
	Psych	Non-psych	p value
Difference between 3-month and pre-op WOMAC score (Median)	27.6	31.6	0.038

The pre-operative EQ-5D was significantly lower in THA psychiatric group compared to controls (0.36 ± 0.26 vs 0.41 ± 0.25, p = 0.011); however, there were no other statistically significant differences in EQ5D between the groups at the 3-month period in either of the THA or TKA subsets. Due to the significant LFR at 1 year for both THA and TKA cohorts, we were unable to validate any outcome analysis for that time period.

Regarding the rest of the secondary outcomes, there was a trend for increased blood transfusion requirements in the TKA psychiatric group compared to the control group (11.2% vs 7.8%, p = 0.057). In addition, the psychiatric THA and TKA groups both had significantly increased LOS compared to their respective control groups. In the THA subset, the psychiatric group had an increased LOS by 1.43 days (p = 0.0028). In the TKA subset, the psychiatric group had an increased LOS by 0.77 days (p = 0.050). Discharge disposition in general was similar between groups, but a sensitivity analysis demonstrated that patients in both the psychiatric THA and TKA groups were discharged home significantly less often, and required a rehabilitation facility, than their respective control groups. In the THA subset, the psychiatric group was discharged home 86.9% of the time compared to the control group at 91.8% (p = 0.024). In the TKA subset, the psychiatric group was discharged home 87.6% of the time compared to the control group at 92% (p = 0.022). There were no other differences between the two group subsets regarding 30-day readmissions, infections, mechanical adverse events and medical adverse events (Tables 3 and 4).

With regard to adverse events, in the THA subset, mechanical (4, 1% vs 9, 2.1%) and medical (2, 0.4% vs 8, 1.9%) adverse events were higher in control group compared to psychiatric group (Table 3). In the TKA subset, mechanical adverse events were similar (1, 0.2%) among psychiatric and control groups. But medical adverse events (13, 2.6% vs 23, 4.6%) were almost doubled in control group compared to psychiatric group (Table 4). However, neither group met statistical significance. Medical and mechanical adverse events were assessed in both groups (Table 6).

**Table 6.** Adverse events.

THA (N = 828)					
Mechanical			Medical		
	Psychiatric diagnosis (n = 414)	No diagnosis (n = 414)		Psychiatric diagnosis (n = 414)	No diagnosis (n = 414)
Complication	0 (0%)	1 (0.2%)	Cerebrovascular accident	0 (0%)	0 (0%)
Hip dislocation	0 (0%)	0 (0%)	Myocardial infarction	1 (0.2%)	2 (0.5%)
Intraoperative fracture	2 (0.5%)	3 (0.7%)	Pulmonary embolism	1 (0.2%)	4 (1.0%)
Post-operative fracture	2 (0.5%)	5 (1.2%)	Deep vein thrombosis	0 (0%)	1 (0.2%)
			Pneumonia	0 (0%)	0 (0%)
			Gastrointestinal bleed	0 (0%)	0 (0%)
			Illeus	0 (0%)	1 (0.2%)
TKA (N = 1000)					
Mechanical			Medical		
	Psychiatric diagnosis (n = 500)	No diagnosis (n = 500)		Psychiatric diagnosis (n = 500)	No diagnosis (n = 500)
Complication	0 (0%)	1 (0.2%)	Cerebrovascular accident	0 (0%)	0 (0%)
Knee dislocation	0 (0%)	0 (0%)	Myocardial infarction	2 (0.4%)	4(0.8%)
Intraoperative fracture	1 (0.2%)	0 (0%)	Pulmonary embolism	8 (1.6%)	15 (3%)
Post-operative fracture	0 (0%)	0 (0%)	Deep vein thrombosis	1 (0.2%)	0 (0%)
			Pneumonia	1 (0.2%)	2 (0.4%)
			Gastrointestinal bleed	1 (0.2%)	0 (0%)
			Illeus	0 (0%)	2 (0.4%)

## Discussion

The primary purpose of this study was to evaluate the role of major psychiatric illness on validated, patient-reported outcomes in TJA. Although the differences in our study groups for the reported scores did not reach the minimal clinically significant differences, psychiatric illness resulted in significantly inferior patient outcomes in the early perioperative period. However, because our sample size was large, the chance that trivial differences impacting our ‘statistical significance’ is low. In other words, the fragility of our p value with the larger sample size is low. There were no major statistical differences in outcomes at 1 year, but we were unable to validate these claims due to the high LFR. In fact, both TKA and THA cohorts had a greater than 90% LFR. This extremely high loss to follow-up highlights a major concern and the need to reinforce the importance of follow-up within patients diagnosed with a psychiatric illness.

Both groups saw significant improvement in functional outcome scores post-operatively for both WOMAC and EQ-5D scores. Regarding our secondary outcomes, patients with psychiatric illness were more likely to have increased LOS and non-routine discharge from hospital. In addition, in the TKA subset with psychiatric illness, there was a trend for increased blood transfusion requirements. There were no other differences in adverse events in either the TKA or THA group.

Although the relationship between psychiatric disorders and clinical outcomes after TJA are yet to be fully elucidated, current literature on the topic largely tends to support our results that psychiatric disease can increase risk of adverse events and result in poor patient outcomes.^19–23^ Buller et al.^22^ in a large retrospective database study demonstrated that patients with a diagnosis of dementia, depression, and schizophrenia had a higher risk of suffering an adverse event post-operatively. In a large retrospective review of the Medicare database, Klement et al.^20^ similarly showed that the presence of a psychiatric disease increased the risk of medical adverse events at the 90-day time period and also increased the risk of periprosthetic fracture, infection, dislocation, and THA revision. Based on these studies, it is clear that patients with a concomitant diagnosis of psychiatric illness are a high-risk group; however, the degree to which these conditions effect clinical outcome are still quite contentious. This is largely due to a number of unique cofounders in this specific population. Psychiatric disease is a complex systemic illness that often leads to poor lifestyle choices, increased comorbidities, as well as stigma and physician bias associated with such a diagnosis.^23^

Our results on LOS and discharge after TJA also correlated well with recent literature. Numerous studies have shown that LOS and rate of non-routine discharge is increased in patients that have psychiatric illness.^22,24^ The reasons for this are likely multifactorial, as patients with mental illness tend to display maladaptive behaviours during rehabilitation. Some of these behaviours include activity limitation, delayed mobilization, and greater dependence on walking aids.^25,26^ In addition, complex pain pathways may make patients with psychiatric illnesses more prone to greater pain perception and delaying mobilization.^27^ These patients are likely to benefit from multidisciplinary care or specialized psychological support. Tristiano et al.^28^ demonstrated that patients who received psychological support through formalized one-on-one programmes after TJA were less likely to be depressed or anxious and were discharged from hospital 1.2 days earlier. From an economic and clinical perspective, the downstream effects of a focused support programme could reduce the total cost of a joint replacement, improve patient outcomes and reduce the loss to follow-up rates.

Our study demonstrated a high LFR among patients with major psychiatric illness undergoing TJA. Among patients in both THA and TKA cohorts, we saw greater than 90% LFR. To our knowledge, this is the highest LFR in the literature that has been identified among patients with major psychiatric illness. In a longitudinal multicentre RCT study assessing follow-up after inpatient versus outpatient psychiatric care, LFR was as high as 46% at 12 months.^29^ This trend is largely supported in the literature, as several studies have demonstrated that psychiatric illness can result in poor compliance and lack of appropriate follow-up.^29–31^ Zhang and Ye^31^ similarly demonstrated a high loss to follow-up of patients with depression being treated in an outpatient setting with nearly 58% LFR at 12 weeks. The majority of the patients lost to follow-up in the aforementioned study were lost primarily after the first 2 weeks, with steady losses seen after that on a biweekly basis, as compared to our study where the majority of patients were lost to follow-up after the 3-month visit. The synergistic effect of poor perioperative outcomes in this population combined with the high loss to follow-up may predispose patients to greater risk of adverse events and poor long-term outcomes. The downstream economic and clinical effects of poor follow-up rate in this population require further attention. Pre-operative psychiatric counselling, a multidisciplinary approach, and access to more mental health support services is necessary to identify at-risk patients to ensure they have appropriate follow-up instructions.

Psychiatric illness is a very broad and encompassing term for a number of heterogeneous conditions. Better metrics and outcomes are also needed to determine how active a particular psychiatric disorder may be at the time of surgery. Halawi et al.^13^ completed a prospective study to determine whether baseline mental health affected outcomes in patients with depression who were undergoing TJA. He found that patients with depression had significant improvements in outcome post-operatively, but these gains were modulated strongly by mental health at the time of the procedure.^21^ There is also likely a complex bidirectional relationship between chronic pain associated with arthritis and psychiatric disorders. Papakostidou et al.^32^ demonstrated that the rate of depression decreased significantly from 42% to 7% within a year of TJA. Similarly, in a Norwegian prescription database study, it was found that hypnotic, analgesic, and anxiolytic medication was significantly decreased after THA.^33^ By treating the mechanical manifestations of arthritis, we may be indirectly treating the psychological manifestations of chronic pain, resulting in a positive synergistic effect on patient outcomes.

The greatest strength of our study was our ability to include a large cohort for each comparison to increase the validity of our results and reduce the fragility of our p value. To our knowledge, this is the largest matched cohort study comparing outcomes among patients with psychiatric illnesses undergoing TJA. However, our study was largely limited by the nature of registry data.

Based on the ABJHI registry, we were unable to categorize results based on the specific type of psychiatric illness. Furthermore, the term psychiatric illness covers a number of heterogeneous conditions that also encompass a wide spectrum of severity. Registry data are well suited to assessing functional outcomes after joint replacement, but due to inherent limitation of retrospective studies, it is difficult to address selection bias. Selection bias by the treating surgeon could have led to selection of patients with less severe or lack of an acute psychiatric illness. Database studies are also restricted by the accuracy of their coding system. Diagnosis coding is a simple and effective way to categorize comorbidities and define study cohorts, but it cannot reflect the acuity or the accuracy of such a diagnosis, especially one as heterogeneous as psychiatric illness. Ideally, a prospective study that captures information on psychiatric illness duration, severity, and treatment would be beneficial. We hope to use the findings of this study to provide the foundation for a prospective study.

## Conclusion

Our results demonstrate that psychiatric illness can result in worse patient reported and clinical outcomes in the early perioperative period after TJA. Patients with psychiatric illnesses can be expected to gain significant improvements in outcome after TJA that are comparable to a baseline population without psychiatric illness. However, patients with psychiatric illnesses are at increased risk of delayed discharge and requirements for a rehabilitation facility prior to being sent home. A high loss to follow-up rate is a major concern in this population, as it may increase sequalae of early adverse events and result in poor long-term outcomes. Evaluation of the type of psychiatric illness, severity, and acuity of the condition should be taken into context. Patients with active or severe psychiatric illness undergoing joint replacement should be referred to their psychiatrist or family physician for optimization of their condition prior to surgery. Furthermore, there should be a greater emphasis on the importance of follow-up in the post-operative period.

## Supplemental Material

sj-pdf-1-smo-10.1177_20503121211012254 – Supplemental material for Do psychiatric disorders affect patient reported outcomes and clinical outcomes post total hip and knee arthroplasty? Click here for additional data file. Supplemental material, sj-pdf-1-smo-10.1177_20503121211012254 for Do psychiatric disorders affect patient reported outcomes and clinical outcomes post total hip and knee arthroplasty? by Sahil Kooner, Jeremy Kubik, Saboura Mahdavi, Sophie (Ghashang) Piroozfar, Hoa Khong, Kanwal Mohan, Eldridge Batuyong and Rajrishi Sharma in SAGE Open Medicine

sj-pdf-2-smo-10.1177_20503121211012254 – Supplemental material for Do psychiatric disorders affect patient reported outcomes and clinical outcomes post total hip and knee arthroplasty? Click here for additional data file. Supplemental material, sj-pdf-2-smo-10.1177_20503121211012254 for Do psychiatric disorders affect patient reported outcomes and clinical outcomes post total hip and knee arthroplasty? by Sahil Kooner, Jeremy Kubik, Saboura Mahdavi, Sophie (Ghashang) Piroozfar, Hoa Khong, Kanwal Mohan, Eldridge Batuyong and Rajrishi Sharma in SAGE Open Medicine

sj-pdf-3-smo-10.1177_20503121211012254 – Supplemental material for Do psychiatric disorders affect patient reported outcomes and clinical outcomes post total hip and knee arthroplasty? Click here for additional data file. Supplemental material, sj-pdf-3-smo-10.1177_20503121211012254 for Do psychiatric disorders affect patient reported outcomes and clinical outcomes post total hip and knee arthroplasty? by Sahil Kooner, Jeremy Kubik, Saboura Mahdavi, Sophie (Ghashang) Piroozfar, Hoa Khong, Kanwal Mohan, Eldridge Batuyong and Rajrishi Sharma in SAGE Open Medicine

sj-pdf-4-smo-10.1177_20503121211012254 – Supplemental material for Do psychiatric disorders affect patient reported outcomes and clinical outcomes post total hip and knee arthroplasty? Click here for additional data file. Supplemental material, sj-pdf-4-smo-10.1177_20503121211012254 for Do psychiatric disorders affect patient reported outcomes and clinical outcomes post total hip and knee arthroplasty? by Sahil Kooner, Jeremy Kubik, Saboura Mahdavi, Sophie (Ghashang) Piroozfar, Hoa Khong, Kanwal Mohan, Eldridge Batuyong and Rajrishi Sharma in SAGE Open Medicine

sj-pdf-5-smo-10.1177_20503121211012254 – Supplemental material for Do psychiatric disorders affect patient reported outcomes and clinical outcomes post total hip and knee arthroplasty? Click here for additional data file. Supplemental material, sj-pdf-5-smo-10.1177_20503121211012254 for Do psychiatric disorders affect patient reported outcomes and clinical outcomes post total hip and knee arthroplasty? by Sahil Kooner, Jeremy Kubik, Saboura Mahdavi, Sophie (Ghashang) Piroozfar, Hoa Khong, Kanwal Mohan, Eldridge Batuyong and Rajrishi Sharma in SAGE Open Medicine

sj-pdf-6-smo-10.1177_20503121211012254 – Supplemental material for Do psychiatric disorders affect patient reported outcomes and clinical outcomes post total hip and knee arthroplasty? Click here for additional data file. Supplemental material, sj-pdf-6-smo-10.1177_20503121211012254 for Do psychiatric disorders affect patient reported outcomes and clinical outcomes post total hip and knee arthroplasty? by Sahil Kooner, Jeremy Kubik, Saboura Mahdavi, Sophie (Ghashang) Piroozfar, Hoa Khong, Kanwal Mohan, Eldridge Batuyong and Rajrishi Sharma in SAGE Open Medicine

sj-pdf-7-smo-10.1177_20503121211012254 – Supplemental material for Do psychiatric disorders affect patient reported outcomes and clinical outcomes post total hip and knee arthroplasty? Click here for additional data file. Supplemental material, sj-pdf-7-smo-10.1177_20503121211012254 for Do psychiatric disorders affect patient reported outcomes and clinical outcomes post total hip and knee arthroplasty? by Sahil Kooner, Jeremy Kubik, Saboura Mahdavi, Sophie (Ghashang) Piroozfar, Hoa Khong, Kanwal Mohan, Eldridge Batuyong and Rajrishi Sharma in SAGE Open Medicine

sj-pdf-8-smo-10.1177_20503121211012254 – Supplemental material for Do psychiatric disorders affect patient reported outcomes and clinical outcomes post total hip and knee arthroplasty? Click here for additional data file. Supplemental material, sj-pdf-8-smo-10.1177_20503121211012254 for Do psychiatric disorders affect patient reported outcomes and clinical outcomes post total hip and knee arthroplasty? by Sahil Kooner, Jeremy Kubik, Saboura Mahdavi, Sophie (Ghashang) Piroozfar, Hoa Khong, Kanwal Mohan, Eldridge Batuyong and Rajrishi Sharma in SAGE Open Medicine

sj-pdf-9-smo-10.1177_20503121211012254 – Supplemental material for Do psychiatric disorders affect patient reported outcomes and clinical outcomes post total hip and knee arthroplasty? Click here for additional data file. Supplemental material, sj-pdf-9-smo-10.1177_20503121211012254 for Do psychiatric disorders affect patient reported outcomes and clinical outcomes post total hip and knee arthroplasty? by Sahil Kooner, Jeremy Kubik, Saboura Mahdavi, Sophie (Ghashang) Piroozfar, Hoa Khong, Kanwal Mohan, Eldridge Batuyong and Rajrishi Sharma in SAGE Open Medicine
