# Supplementary material for: Do psychiatric disorders affect patient reported outcomes and clinical outcomes post total hip and knee arthroplasty?
Source: SAGE Open Med. 2021 Apr 29;9:20503121211012254. doi: 10.1177/20503121211012254 (PMC8107666; doi:10.1177/20503121211012254)
Supplement: sj-pdf-1-smo-10.1177_20503121211012254 – Supplemental material for Do psychiatric disorders affect patient reported outcomes and clinical outcomes post total hip and knee arthroplasty? [file sj-pdf-1-smo-10.1177_20503121211012254.pdf]

Outcome

Please complete the survey below.

Thank you!

The following questions concern the amount of PAIN you have experienced in your HIP or KNEE joint. For each situation please choose the amount of PAIN experienced in the last 48 hours.How much pain have you had...

		None	Mild	Moderate	Severe	Extreme
1)	when walking on a flat surface?	○	○	○	○	○
2)	when going up or down stairs?	○	○	○	○	○
3)	at night while in bed? (that is ○ that disturbs your sleep).	○	○	○	○	
4)	when sitting or lying?	○	○	○	○	○
5)	when standing?	○	○	○	○	○

Page 2 of 6 - Womac Stiffness

Think about the STIFFNESS (not pain) you felt during the last 48 hours caused by the arthritis in your hip or knee joint. STIFFNESS is a sensation of decreased ease in moving your joint.How severe has your stiffness been...

		None	Mild	Moderate	Severe	Extreme
6)	after you first woke up in the ○ morning?	○	○	○	○	
7)	after sitting or lying down or ○ while resting later in the day?	○	○	○	○	

Page 3 of 6 - Womac Difficulty (1 of 3)

CURRENT ACTIVITIES: DEGREE OF DIFFICULTY

		None	Mild	Moderate	Severe	Extreme
8)	Descending stairs	○	○	○	○	○
9)	Ascending stairs	○	○	○	○	○
10)	Rising from sitting	○	○	○	○	○
11)	Standing	○	○	○	○	○
12)	Bending to the floor	○	○	○	○	○
13)	Walking on a flat surface	○	○	○	○	○

Page 4 of 6 - Womac Difficulty (2 of 3)

CURRENT ACTIVITIES: DEGREE OF DIFFICULTY

		None	Mild	Moderate	Severe	Extreme
14)	Getting in/out of a car	○	○	○	○	○
15)	Shopping	○	○	○	○	

○

16)

	Putting on your socks/stockings	◯	◯	◯	◯	◯
17)	Rising from bed.	◯	◯	◯	◯	◯
18)	Taking off socks/stockings	◯	◯	◯	◯	◯
19)	Lying in bed	◯	◯	◯	◯	◯

Page 5 of 6 - Womac Difficulty (3 of 3)

CURRENT ACTIVITIES: DEGREE OF DIFFICULTY

		None	Mild	Moderate	Severe	Extreme
20)	Getting in/out of bath/shower	◯	◯	◯	◯	◯
21)	Sitting	◯	◯	◯	◯	◯
22)	Getting on or off the toilet	◯	◯	◯	◯	◯
23)	Heavy domestic duties	◯	◯	◯	◯	◯
24)	Light domestic duties	◯	◯	◯	◯	◯

Page 6 of 6 - EQ5D

Under each heading, please pick the ONE box that best describes your health TODAY.

25) Mobility

◯ I have no problems in walking about
◯ I have slight problems in walking about
◯ I have moderate problems in walking about
◯ I have severe problems in walking about
◯ I am unable to walk about

26) Self-Care

◯ I have no problems washing or dressing myself
◯ I have slight problems washing or dressing myself
◯ I have moderate problems washing or dressing myself
◯ I have severe problems washing or dressing myself
◯ I am unable to wash or dress myself

27) Usual Activities (e.g. work, study, housework, family or leisure activities)

◯ I have no problems doing my usual activities
◯ I have slight problems doing my usual activities
◯ I have moderate problems doing my usual activities
◯ I have severe problems doing my usual activities
◯ I have I am unable to do my usual activities

28) Pain/Discomfort

◯ I have no pain or discomfort
◯ I have slight pain or discomfort
◯ I have moderate pain or discomfort
◯ I have severe pain or discomfort
◯ I have extreme pain or discomfort

29) Anxiety/Depression

◯ I am not anxious or depressed
◯ I am slightly anxious or depressed
◯ I am moderately anxious or depressed
◯ I am severely anxious or depressed
◯ I am extremely anxious or depressed
